# Development stage-specific proteomic profiling uncovers small, lineage specific proteins most abundant in the *Aspergillus Fumigatus* conidial proteome

**DOI:** 10.1186/1477-5956-10-30

**Published:** 2012-04-30

**Authors:** Moo-Jin Suh, Natalie D Fedorova, Steven E Cagas, Susan Hastings, Robert D Fleischmann, Scott N Peterson, David S Perlin, William C Nierman, Rembert Pieper, Michelle Momany

**Affiliations:** 1 The J. Craig Venter Institute, 9704 Medical Center Drive, Rockville, MD, USA; 2 University of Medicine and Dentistry of New Jersey, Newark, NJ, USA; 3 Department of Plant Biology, University of Georgia, Athens, GA, USA

**Keywords:** Mass spectrometry, LC-MS/MS, APEX, Shotgun proteomics, *Aspergillus fumigatus*, Germination, Spore, Conidia, Fungi, Hypothetical proteins

## Abstract

**Background:**

The pathogenic mold *Aspergillus fumigatus* is the most frequent infectious cause of death in severely immunocompromised individuals such as leukemia and bone marrow transplant patients. Germination of inhaled conidia (asexual spores) in the host is critical for the initiation of infection, but little is known about the underlying mechanisms of this process.

**Results:**

To gain insights into early germination events and facilitate the identification of potential stage-specific biomarkers and vaccine candidates, we have used quantitative shotgun proteomics to elucidate patterns of protein abundance changes during early fungal development. Four different stages were examined: dormant conidia, isotropically expanding conidia, hyphae in which germ tube emergence has just begun, and pre-septation hyphae. To enrich for glycan-linked cell wall proteins we used an alkaline cell extraction method. Shotgun proteomic resulted in the identification of 375 unique gene products with high confidence, with no evidence for enrichment of cell wall-immobilized and secreted proteins. The most interesting discovery was the identification of 52 proteins enriched in dormant conidia including 28 proteins that have never been detected in the *A. fumigatus* conidial proteome such as signaling protein Pil1, chaperones BipA and calnexin, and transcription factor HapB. Additionally we found many small, Aspergillus specific proteins of unknown function including 17 hypothetical proteins. Thus, the most abundant protein, Grg1 (AFUA_5G14210), was also one of the smallest proteins detected in this study (M.W. 7,367). Among previously characterized proteins were melanin pigment and pseurotin A biosynthesis enzymes, histones H3 and H4.1, and other proteins involved in conidiation and response to oxidative or hypoxic stress. In contrast, expanding conidia, hyphae with early germ tubes, and pre-septation hyphae samples were enriched for proteins responsible for housekeeping functions, particularly translation, respiratory metabolism, amino acid and carbohydrate biosynthesis, and the tricarboxylic acid cycle.

**Conclusions:**

The observed temporal expression patterns suggest that the *A. fumigatus* conidia are dominated by small, lineage-specific proteins. Some of them may play key roles in host-pathogen interactions, signal transduction during conidial germination, or survival in hostile environments.

## Background

*Aspergillus fumigatus* is the most common airborne fungal pathogen, which can infect ever increasing numbers of patients with lung disease, immune system disorders or undergoing immunosuppression therapy [1]. In patients with asthma and cystic fibrosis, it can cause allergic diseases like allergic bronchopulmonary aspergillosis. In immunosuppressed individuals such as leukemia and bone marrow transplant patients, inhalation of *A. fumigatus* conidia (asexual spores) can cause invasive aspergillosis (IA), a life-threatening disease, which is difficult to diagnose and treat. If successful in reaching the innate immune defense in the lungs, conidia germinate into hyphae, long finger-like projections that invade host tissues and blood vessels within days or even hours after colonization. Despite the importance of the early morphogenetic transition for initiation of infection, its specific mechanisms are not all well-understood, which hinders the development of better diagnostic and therapeutic approaches to combat IA.

The availability of two sequenced *A. fumigatus* genomes, AF293 and A1163 [2,3], have enabled high-throughput transcriptomic and proteomic approaches and, thus, greatly facilitated the pace of discovery of new biomarkers and therapeutic targets for IA. Previous proteomic studies have identified a number of proteins involved in early stages of *A. fumigatus* development and early interactions with the human host [4]. Traditionally proteomic studies rely on gel-based separations such as 2-dimensional polyacrylamide gel electrophoresis (2-D PAGE) followed by mass spectrometry (MS). The methods helped to identify reactive oxygen detoxification enzymes, pigment biosynthesis enzymes and other highly abundant proteins in the *A. fumigatus* conidial proteome [5-8].

While 2D gel approaches can identify proteins in their intact forms, they lack sufficient sensitivity and dynamic range for protein quantification. Furthermore, 2D gels regularly fail to resolve proteins with physicochemical characteristics such as high hydrophobicity, extreme p*I* and M_r_ values and covalent attachment to membranes or cell walls [9]. As a result, little is known about expression status and functional roles of such proteins*.* Quantitative shotgun proteomics based on liquid chromatography tandem MS (LC-MS/MS) holds promise to more comprehensive proteome surveys, including comparative analyses of proteins from different developmental stages [10]. Recently, we (SC and DP) profiled *A. fumigatus* early development proteome states using shotgun proteomics based on isobaric tagging of peptides (iTRAQ), accompanied by a simultaneous transcriptome analysis [11]. This approach resulted in identification of 231 proteins with high confidence. The current study also aims to survey the *A. fumigatus* early developmental proteome, although it is focused on earlier time points and involves different growth conditions. Although the hydrophobin RodA was among the most abundant proteins, the attempt to enrich for cell wall-immobilized proteins was apparently not successful. Using a shotgun proteomics approach, 375 proteins were identified including 207 proteins that have not been detected using iTRAQ shotgun proteomics [11]. Additionally we found 28 dormant conidia-enriched proteins that have not been previously detected in the *A. fumigatus* proteomes of conidia or pre-septation hyphae.

## Results and discussion

### Selection of time points that represent distinct stages of early fungal development for proteomics analysis

In most fungi, conidial dormancy is controlled by exogenous factors such as the availability of moisture, oxygen and nutrients [12]. It has been established that, when inoculated to culture medium containing a carbon source, *A. fumigatus* conidia synchronously break dormancy and begin nuclear division and morphological development. Nuclear division and morphological development remain roughly synchronous for at least 12 h, a time that encompasses the first several rounds of mitosis and early developmental landmarks [13]. In this study, we exploited this inherent synchrony to characterize the *A. fumigatus* proteome during the early stages of fungal development.

**Figure 1:**
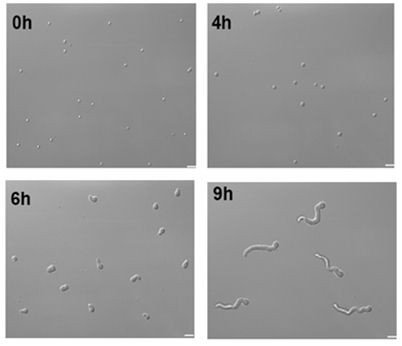
***A. fumigatus*early development stages selected for proteomic analysis.** For the 0 h time point dormant conidia were stained with Calcofluor and Hoechsts. For the 4, 6 and 9 h time points, 3 x 10^6^ spores were inoculated into 10 mL of GMM and incubated at 37°C. The samples were fixed and stained with Calcofluor and Hoechsts. Upper panel shows DIC image lower panel shows stained florescent image. All pictures were taken at 100X magnification. Scale bar = 3 μm.

To select specific stages for proteomics analysis, *A. fumigatus* conidia were inoculated in glucose complete medium and sequential samples were examined microscopically for developmental landmarks every 30 min. As summarized in Figure 1, the conidium expands isotropically for 4 h at 37°C in complete medium. Most cells polarize and send out the first germ tube between 5 and 6 h and continue to elongate becoming hyphae. The first septum forms near the base of the hypha between 9 and 10 h, asymmetrically dividing the hypha into two compartments. At about the same time, the first branch forms on the apical side of the septum. Hyphae continue to elongate and branch and eventually form a mycelial mat. Based on the microscopy data, four time points were selected for proteomics analysis: dormant conidia (0 h), isotropically expanding conidia (4 h), hyphae with early germ tubes (6 h), and pre-septation hyphae (8 h).

Although *A. fumigatus* conidia were grown *in vitro*, we believe that these four time points represent critical developmentally-matched stages of fungal growth *in vivo*. Previous work has shown that the cell wall of *A. fumigatus* is organized in domains that change during early growth [14]. It is likely that some of this change is associated with new proteins being added to the wall at different stages of development as well as by reorganization and modification of proteins within the wall. Though the timeline of *A. fumigatus* development within human hosts is not known, we can extrapolate from the *in vitro* data cited above and in mouse model systems [15].

### *A. Fumigatus* proteins expressed during early fungal development

**Figure 2:**
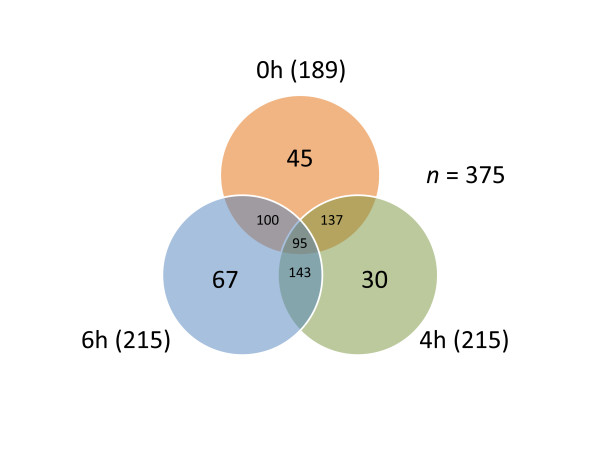
**Venn diagram of proteins detected at 0 h, 4 h and 6 h of fungal growth.** Each circle represents the number of proteins detected with APEX expression values above 3,500 at different time points. The numbers of analyzed and detected proteins for each time point are shown in Table 1.

*A. fumigatus* proteins were extracted using a mild alkali method. They were analyzed using LC-MS/MS followed by a modified spectral counting technique, called APEX [16]. Using this approach, we detected 570 unique proteins which represented 5.6% of the predicted *A. fumigatus* proteome. The estimated sequence coverage for proteins ranged from 4% to 100%, and Mascot scores ranged from 40 to 4,978. Theoretical isoelectric points (p*I*s) varied between 3.8 and 11.8, and molecular masses (M_r_) between 6,287 and 234,143 Da. Experimentally observed proteins were mapped against the theoretical proteome in relation to M_r_ and p*I* (Additional file 1). Of the observed proteins, 14.4% were acid (p*I* below 5) and 26.4% basic proteins (p*I* above 9). In addition, 27.5% of the proteins had molecular masses of less than 20 kDa. The hydrophobicity of the proteins was calculated using the GRAVY index, an arbitrary threshold for high hydrophobicity, and only four proteins had values above 0.2. These result imply that our extraction technique was somewhat biased against highly hydrophobic proteins. In Additional file 2, the proteins and all of the respective identified tryptic peptides are listed. Due to quantitative variability of spectral counting methods in the low abundance range [17], only proteins with an APEX value of 3,500 or higher in at least one time point were analyzed further (Additional file 3). This approach resulted in the identification of 375 proteins including 189, 215, 215 and 230 proteins detected at the respective four time points (0 h, 4 h, 6 h, and 8 h) (Table 1 and Figure 2). While low abundance proteins are of interest, they are notoriously difficult to quantify reliably with shotgun proteomics approaches on most instrument platforms. The newest LC-MS platforms, e.g. the LTQ Orbitrap Velos, promise to eliminate these bottlenecks for proteome-wide quantification [18,19].

**Table 1:** **Proteins expressed during early development in*A. fumigatus***

**Time points**	**Matched peptides**	**Proteins detected**	**FDR (%)**	**Proteins with APEX >3,500**	**Enriched proteins**
0-8 h	n/a	n/a	n/a	143	38*
0 h	3148	325	2.19	189	52
4 h	2261	299	2.30	215	85
6 h	2657	319	1.17	215	127
8 h	3615	361	0.64	230	119
Total	11681	570	1.60	375	n/a

Table legend: *Constitutively expressed proteins at all four time points.

**Figure 3:**
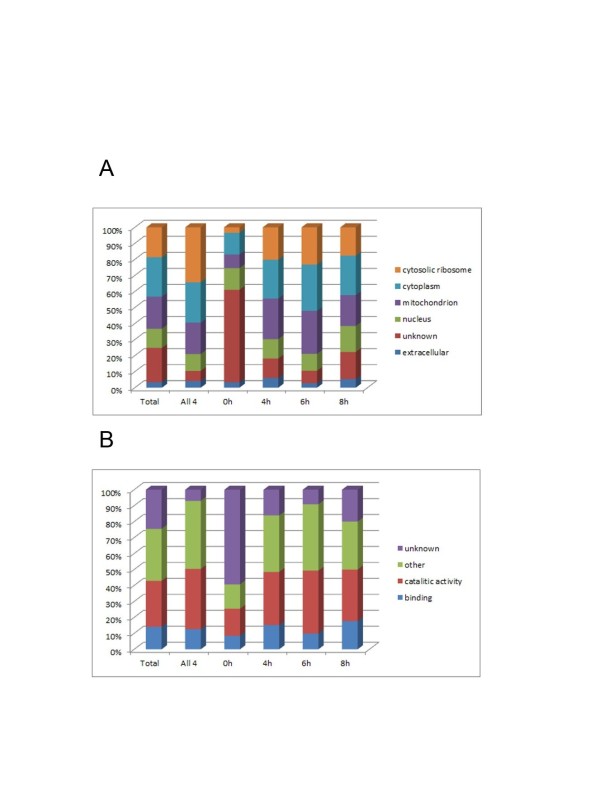
**Cellular localizations and molecular functions of proteins enriched during early fungal development.** Gene Ontology (GO) Slim terms were generated from general GO terms as described in Methods. (**A**) Cellular localizations; (**B**) Molecular functions.

About 83% of the 375 proteins analyzed had at least one assigned Gene Ontology (GO) term from the Biological Process, Molecular Function, and Cellular Component ontologies (Figure 3 and Additional file 4 and Additional file 5). Most proteins were intracellular including 75 mitochondrial, 92 cytoplasmic, 70 ribosomal, and 45 nuclear proteins. Only thirteen of the 375 proteins were previously associated with cell wall, plasma membrane or extracellular regions based on their glycosylphosphatidyl-inositol (GPI) anchor motif or experimental evidence. The large fraction of intracellular proteins was unexpected, since we applied a mild alkali extraction method [20] that was previously shown to release alkali-sensitive proteins covalently linked to glucans in *Candida albicans* cell walls and also to recover proteins released from lysed cells but retained in the cell pellets in insoluble or cell surface-bound forms. It remains to be shown that *A. fumigatus* immobilizes proteins in its cell wall via glycan linkages. Despite the apparent lack of enrichment for cell wall proteins, we detected 18 out of 62 proteins previously associated with the secreted *A. fumigatus* proteome [21] (Table 2). Notably, fewer than 6% (22 proteins) detected in this study had a predicted signal peptide or signal anchor sequence. Also, only 20 (3%) putative integral membrane proteins were identified; all present at very low levels. This was less than expected based on the total number of putative proteins in the *A. fumigatus* proteome [2,3], suggesting that extraction of fungal membrane proteins for proteomics analysis remains a difficult task.

**Table 2:** Comparisons with other early development proteomics studies

**Other studies**	**Proteins enriched at specific time points in this study**						**Time point**	**Method used**	**Reference**
**Proteins detected**	**Total(375)**	**0-8 h(143)**	**0 h(52)**	**4 h (85)**	**6 h (127)**	**8 h (119)**			
25	14	9	4	6	0	1	0 h	2D-Gel	Asif 2006
231	168	99	6	46	78	63	0-16 h	MALDI-MS/iTRAQ	Cagas 2011
57	37	23	2	15	15	13	8 h	iTRAQ	Cagas 2011
34	23	10	2	6	13	8	16 h	iTRAQ	Cagas 2011
61	42	30	1	14	23	16	4 h	2D-Gel	Singh 2010
63	25	17	9	5	5	3	0 h	2D-Gel	Teutschbein 2010

Table legend: proteins enriched with log2 ratios of expression values > 2.

Most proteins were involved in translation, respiratory metabolism, amino acid and carbohydrate biosynthesis, tricarboxylic acid cycle, and other housekeeping functions. Five common allergens, Asp F3, F8, F9, F12 and F22, and three adhesin-like proteins were detected. We also found four known virulence factors including cell wall organization protein Ecm33 (AFUA_4G06820), Mn superoxide dismutase SodB (AFUA_4G11580), homocitrate synthase HcsA (AFUA_4G10460), and citrate synthase Cit1/McsA (AFUA_6G03590). One protein, conidial pigment biosynthesis scytalone dehydratase Arp1 (AFUA_2G17580), was implicated in interactions with the host.

Comparisons with previous studies of the early *A. fumigatus* proteome showed that different shotgun approaches complement each other with respect to protein identification (Table 2). We detected 14 out of 26 conidial surface associated proteins [5] and 28 out of 40 most abundant intracellular conidial proteins [6] previously found by 2-D PAGE. Additionally, we found 55 out of 66 immuno-reactive cytosolic proteins extracted from germinating conidia [8]. Our study also identified 168 out of 231 proteins previously detected in *A. fumigatus* during early development by iTRAQ [11]. Further comparison with the Cagas et al. study showed that quantification of expression values using shotgun proteomics methods continues to be a challenge (see below). Some of these discrepancies can be explained by different time points or score cutoffs used to define differentially expressed genes and proteins, while others may result from differences in the proteomics approaches or growth media used.

### Proteins expressed at all four stages during early fungal development

Out of 375 proteins, 143 were expressed at all four time points, while the remaining 232 were not detected at one or more time points (Additional file 6). All but ten proteins had an assigned GO biological function (Figure 3). One third of the proteins were ribosomal components or related proteins that function in translation. The rest had an assigned role in oxidative phosphorylation, amino acid biosynthesis, gluconeogenesis, and tricarboxylic acid cycle. All 143 proteins have orthologs in other *Aspergillus* species, and the majority of them are highly evolutionarily conserved across a broad range of fungal species. All but twenty proteins were encoded in central regions of chromosomes (i.e. least 600 Kb from telomeres), which typically are reserved for the most evolutionary conserved functions such as genome replication, expression, and central metabolism. Most of the 143 proteins were detected in *A. fumigatus* in earlier proteomics studies (Table 2). Thus, 70% of them were previously identified using iTRAQ proteomics [11].

Most proteins that were expressed at all four time points showed a moderate increase in abundance at 4 h, 6 h and 8 h with respect to the 0 h time point. The most abundant proteins detected at all four time points included conidial hydrophobin Hyp1/RodA (AFUA_5G09580), allergens Asp F3 (AFUA_6G02280), Asp F8 (AFUA_2G10100) and Asp F22 (AFU A_6G06770), and subunits of the translation elongation factor. Out of 143 proteins, 38 were constitutively expressed at all four time points. These were defined as proteins with log2 ratios less than 1.5 (see Methods). Four of these constitutively expressed proteins were characterized as upexpressed in conidia using iTRAQ or 2-D PAGE approaches [6,11]. Thus, allergen Asp F22 (AFUA_6G06770), Hyp1/RodA (AFUA_5G09580), malate dehydrogenase (AFUA_6G05210), and zinc-containing alcohol dehydrogenase (AFUA_4G08240) were previously characterized as conidia-enriched proteins in both of these studies. In contrast, our analysis showed only a very moderate decrease in their abundance levels at 4 h or at later stages (Additional file 6). We limited the differential expression analysis comparing developmental time points to proteins that had at least 4 significant peptides from Peptide/ProteinProphet analysis at a 5% false discovery rate set.

### Dormant conidia enriched proteins (0 h)

**Figure 4:**
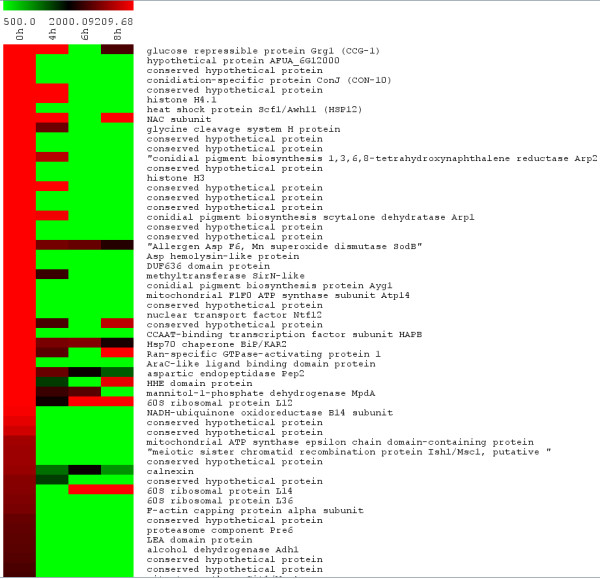
**Proteins of high abundance in*A. fumigatus*conidia.** Abundances derived from APEX values ranging from o to 440,000 are displayed in a heat map generated with the MeV analysis software. More protein information is provided in Additional file 7 where proteins are listed in the same order.

To identify proteins enriched at 0 h in comparison to 4 h in *A. fumigatus*, we analyzed all proteins expressed at 0 h with an APEX score above 3,500. Using a cutoff of less or equal than −1.5 for log2 expression ratios (4 h/0 h), 52 dormant conidia-enriched proteins were found. Most of these proteins were not detected at 4 h, 6 h and 8 h (Figure 4). Half of the conidia enriched proteins have no assigned biological function (Figure 3 and Additional file 7), including 17 ‘hypothetical proteins’. The rest tend to be involved in sporulation, response to oxidative and hypoxic stress, cell wall biosynthesis, and secondary metabolite biosynthesis. Only one third of the 0 h enriched proteins have no homologs in other fungi besides the two closest relatives of *A. fumigatus*: *Aspergillus clavatus* and *Neosartoria fischeri* (*Aspergillus fischerianus*). This is consistent with previous findings that most conidia enriched transcripts have no assigned biological roles and are lineage specific in other fungi (see [22] for review). Interestingly, small proteins were significantly over-represented in dormant conidia. Thus, the average M.W. of these proteins was 26,294, which was almost half the average M.W. of the proteins enriched at the 8 h time point (44,256).

Out of 52 proteins, 28 have never been previously identified as abundant or over-represented in *A. fumigatus* dormant conidia [5,6,8,11]. Using the WoLF PSORT software tool, only two functionally not characterized proteins (AFUA_1G13670 and GPI-anchored protein AFUA_4G09600) were predicted to localize extracellularly (Additional file 7). The smallest and most abundant protein detected at 0 h was a protein of unknown function called Grg1 (AFUA_5G14210). Although Grg1 has not been identified in previous proteomics studies, its transcripts have been detected in *A. fumigatus* conidia [23] and shown to be up-regulated in conidia exposed to neutrophils [24]. In *A. nidulans*, Grg1 transcripts are up-regulated in mycelia exposed to light [25]. Its orthologs in other fungi have been proposed to function as a developmentally regulated, general stress protein involved in lifespan control [26,27].

Another interesting protein of unknown function enriched at 0 h was ConJ (AFUA_6G03210). Although ConJ’s biological role is unknown, its transcripts were shown to be upregulated in the early *A. fumigatus* transcriptome [11] and during initiation of murine infection [15]. Its ortholog, CON-10, was associated with conidial development in *N. crassa*. Transcripts of CON-10 were shown to accumulate in vegetative mycelia upon blue light exposure and during conidial development [28]. Both Grg1 and ConJ were computationally predicted to have nuclear localization. Another 0 h-enriched protein of note was the pigment biosynthesis scytalone dehydratase Arp1 (AFUA_2G17580). Arp1 is encoded by the six-gene pigment biosynthesis cluster, which also encodes proteins that have been earlier associated with conidia. The conidial pigment, melanin, has been shown to contribute to fungal virulence in a murine model [29] and to modulate the host cytokine response by masking specific ligands on the *A. fumigatus* cell surface [30,31]. 1,8-dihydroxynaphthalene-melanin was shown to inhibit phagolysosomal acidification [32].

A few heat shock proteins and other chaperons involved in maturation of protein complexes were also upexpressed in the *A. fumigatus* dormant conidia. Many of these proteins have never been previously associated with *A. fumigatus* spores including heat shock protein Scf1/Awh11 (AFUA_1G17370), nascent polypeptide-associated complex subunit Egd2 (AFUA_6G03820), calnexin ClxA (AFUA_4G12850), and Hsp70 chaperone BipA (AFUA_2G04620). The exact biological role of Scf1 is unknown. It is a possible target of transcription factor CrzA, which is a downstream effector of the calcineurin signaling pathway and regulates conidial germination, hyphal growth, and pathogenesis in *A. fumigatus*[33,34]. In *A. nidulans*, Scf1 transcription is repressed by StuA*,* which also regulates multicellular complexity during asexual reproduction, ascosporogenesis and multicellular development during sexual reproduction [35]. Scf1 also has a *S. cerevisiae* ortholog, HSP12, which is a plasma membrane protein involved in maintenance of membrane organization under stress and in response to heat shock, oxidative stress, and osmotic stress [36].

Similarly, chaperons ClxA and BipA are involved in unfolded protein response and possibly ER stress in fungi. In filamentous fungi, calnexin is involved in N-glycan-dependent quality control of folding of cell-wall-targeted glycoproteins [37,38]. Glycosylation is a conserved posttranslational modification that is essential for cell wall function [39]. A recent 2-D PAGE study showed that overexpression of calnexin and a putative HSP70 chaperone is activated by the deletion of the *cwh*41 gene encoding glucosidase I in *A. fumigatus*, which also leads to ER stress and possibly activates the ER-associated degradation [40].

Among other unusual findings was the detection of a putative transcription factor, HapB (AFUA_2G14720) and histones H3 and H4.1 (AFUA_1G13780 and AFUA_1G13790). Orthologs of HapB have been shown to function in regulation of carbohydrate metabolism in *A. nidulans*[41] and sporulation in yeast [42], and the subunit HapE of the CCAAT-binding complex was previously identified in dormant conidia [6]). This complex was shown to be a key regulator of redox homeostasis in *A. nidulans*[43]. Histones H3 and H4.1 have been implicated in sporulation in *S. cerevisiae*[44].

In addition to 28 novel conidia enriched proteins, 24 proteins including known virulence factors were discovered in previous proteomics studies (Table 2) [5,6,8,11]. Nine of the 0 h-enriched *A. fumigatus* proteins were identified as overexpressed in dormant conidia vs. mycelium by Teutschbein and colleagues using 2-D PAGE [6]. Among these were Mn superoxide dismutase SodB (AFUA_4G11580) and endopeptidase Pep2 (AFUA_3G11400), two conidial pigment biosynthesis proteins, Ayg1 and Arp2 (AFUA_2G17550 and AFUA_2G17560), a putative methyltransferase (AFUA_8G00550), and 2-methylcitrate synthase McsA (AFUA_6G03590). SodB, also known as allergen Asp F6, was also detected in the secreted *A. fumigatus* proteome [21]. SodB is considered a putative virulence factor, because it detoxifies superoxide anions and its transcripts are up-regulated in conidia exposed to neutrophils and by the oxidative agent menadione [24]. However, a triple deletion mutant (*sod1sod2sod3*) did not show attenuation in virulence[45]. Endopeptidase Pep2 is a conidia surface-associated protein [5], whose transcripts are up-regulated in conidia exposed to neutrophils [5,6,24,46]. Alb1, not identified in this study, and McsA have been characterized putative virulence factors in *A. fumigatus*. The *alb1* gene is also involved in conidial morphology and resistance to oxidative stress [47]. AFUA_8G00550 is encoded by a pseurotin A biosynthesis cluster [48]. It is induced during hypoxia and over-represented in conidial [6,49].

Additionally, three of 0 h-enriched proteins were previously identified as highly abundant in the conidial proteome by the same authors [6]. The list includes a hypothetical protein (AFUA_6G12000), an Asp hemolysin-like protein (AFUA_4G02805), and mannitol-1-phosphate dehydrogenase MpdA (AFUA_2G10660). AFUA_6G12000 is the second most abundant protein at 0 h and is unique to *A. fumigatus* and its close relative, *N. fischeri.* The functional role of Asp hemolysin-like protein (AFUA_4G02805) is not yet known. Its paralog, Asp hemolysin (AFUA_3G00590), was recently identified as a major secreted protein expressed in resting and germinating conidia and during hyphal development [21]. Both proteins belong to the protein family of aegerolysins, which includes a large number of bacterial and fungal proteins that function in sporulation and development. MpdA protein is induced by heat shock and reacts with rabbit immunosera exposed to *A. fumigatus* germling hyphae [50]. In *A. niger*, the *mpdA* gene expression is increased in the sporulating mycelium [51,52]. This indicates that mannitol biosynthesis may be developmentally regulated in aspergilli. Mannitol itself has been shown to play a key role in ensuring the stress tolerance of *A. niger* conidiospores [52].

### Expanding conidia-enriched proteins (4 h)

Out of 215 proteins detected at the 4 h time point, 85 were identified as up-expressed at 4 h in comparison to 0 h in *A. fumigatus* conidia. Remarkably, 25 of these proteins (29%) were not detected in dormant conidia, while 44 (52%) were also up-expressed at 6 h and 8 h in comparison to 0 h. This is consistent with the view that the dramatic shift in protein expression associated with conidial expansion happens between 0 h and 4 h time points. Most proteins (85%) had an assigned GO biological function, with translation and tricarboxylic acid cycle being the most common ones (Additional file 8). Almost half of 4 h enriched proteins (52 out of 85 proteins) were previously identified in the *A. fumigatus* conidial proteome [5,6,8,11] (Table 2).

While the majority of 4 h enriched proteins were intracellular, the list also includes five cell wall proteins such as cell wall organization protein Ecm33 (AFUA_4G06820), which was earlier implicated in conidial germination, antifungal drug resistance, and hypervirulence [53,54]. Four GPI-anchored proteins were also identified including beta-1,3-endoglucanase EglC (AFUA_3G00270), which has been implicated in cell wall organization and biosynthesis [55]. EglC was also detected in the *A. fumigatus* immunosecretome, secreted proteome and in germinating conidia [8,21,56].

Among the most abundant proteins in expanding conidia were allergens Asp F8/60 S ribosomal protein P2 (AFUA_2G10100) and Asp F3 (AFUA_6G02280), several cytosolic ribosomal subunits, and the putative cell cycle regulator Wos2 (AFUA_5G13920). *A. fumigatus* Wos2 has been shown to be recognized by immunosera from rabbits exposed to conidia [50], while its orthologs function in regulation of the cell cycle in *A. niger* and of telomerase activity in yeast [57,58]. The predominance of known allergens and other immunoreactive proteins in expanding conidia is consistent with previous studies. This initial stage of spore germination, also known as “swelling,” triggers the recruitment of host inflammatory cells.

Another immunoreactive protein enriched at 4 h was CipC (AFUA_5G09330), which was shown to react with immunosera from rabbits exposed to *A. fumigatus* conidia [50]. It has never before been associated with the conidial proteome, but described a major hyphal-specific protein [59]. Proteomic evidence indicated that CipC is a secreted protein [5]. Its exact function is unknown, although it was suggested that it is involved in competitive interactions between bacteria and aspergilli. CipC was associated with the hyphal morphotype that enables invasive growth during infection. Proteome analysis of *A. nidulans* identified its close homolog CipC (but not AFUA_5G09330) as a protein associated with the response to stress and the antibiotic concanamycin A [60].

Amino acid biosynthesis proteins were also abundant at 4 h including homocitrate synthase HcsA (AFUA_4G10460), which has been implicated in *A. fumigatus* virulence and is considered a possible antifungal drug target [61]. HcsA is also expressed at 6 h and 8 h. The protein is required for lysine biosynthesis and has been shown to be induced by heat shock [62]. This virulence factor has not been associated previously with *A. fumigatus* conidial proteome, however, its transcript is known to be highly induced during conidial germination [12].

Among unusual findings was the discovery of regulatory protein suAprgA1 (AFUA_3G09030), which has not been previously associated with conidia. Although its exact function is unknown, it is a highly conserved protein with putative homologs in mammals, fungi and protozoa. Its orthologs have been shown to function in aerobic respiration in *S. cerevisiae* and in regulation of penicillin biosynthesis in *Aspergillus nidulans*[63]. In contrast, its homolog regulates the RNA-binding activity of a protein that guides RNAs during the mitochondrial RNA editing process in *Trypanosoma brucei*[64].

Additionally, some proteins were detected at 0 h and 4 h time points such as cell wall integrity signaling protein Pil1 (AFUA_6G07520). It is the only one signaling protein detected in the early *A. fumigatus* proteome. Its ortholog has been detected in the *A. nidulans* proteome at 0 h and 1 h time points [65]. It localized to the conidial periphery and in punctate structures in mycelia [65,66]. *A. fumigatus* Pil1 is similar to yeast sphingolipid long chain base-responsive protein PIL1, which is a primary component of large immobile cell cortex structures associated with endocytosis. PIL1 null mutants show activation of Pkc1p/Ypk1p stress resistance pathways in *S. cerevisiae*[67].

### Early germ tube-enriched proteins (6 h)

Out of 215 proteins found in hyphae with early germ tubes, 127 (59%) were identified as over-expressed in comparison to dormant conidia in *A. fumigatus*. The vast majority (94%) of 6 h enriched proteins had an assigned GO biological function (Additional file 9). Most common functions included translation, ATP synthesis coupled electron transport, amino acid biosynthesis, gluconeogenesis, and tricarboxylic acid cycle. Almost half were ribosomal components and proteins that function in translation. The proteins appear to be evolutionarily conserved across a broad range of fungal species as well as in other eukaryotes including humans. All but four proteins of the 127 proteins (97%) were encoded by genes located in central regions of chromosomes (>300 Kb from telomeres), which on average harbor only 85% of *A. fumigatus* genes. Only two proteins were annotated as “hypotheticals”, because they shared no sequence similarity with any characterized protein or domain in public databases. Both proteins were only detected at 6 h.

Eighty five of the 127 proteins (67%) were also enriched in early germ tubes in comparison to expanding conidia, reflecting continuous exponential increase in the biosynthetic capacity during these three developmental stages. The most abundant proteins among those were translation elongation factor subunits, components of the cytosolic ribosome, thiazole biosynthesis enzyme ThiF (AFUA_6G08360), glyceraldehyde 3-phosphate dehydrogenase GpdA (AFUA_5G01970), and plasma membrane H + −ATPase Pma1 (AFUA_3G07640). ThiF has not been detected in the *A. fumigatus* proteome prior to this study. The ThiF yeast ortholog, THI4, has been shown to catalyze formation of a thiazole intermediate during thiamine biosynthesis and to be required for mitochondrial genome stability in response to DNA damaging agents [68]. GpdA has been shown to react with immunosera from rabbits exposed to *A. fumigatus* conidia [50].

Some of the 6 h enriched proteins may have important roles in establishing mammalian infection. Thus homocitrate synthase HcsA (AFUA_4G10460) and superoxide dismutase SodA (AFUA_5G09240), previously implicated in the initiation of infection, are up-expressed at this stage. HcsA has not been associated with *A. fumigatus* conidia or germling hyphae in proteomics studies. Furthermore, transcripts for six of these proteins were up-regulated in *A. fumigatus* germlings during initiation of murine infection [15]. The list includes ThiF, mentioned above, cell wall glucanase BtgE (AFUA_8G05610), superoxide dismutase SodA (AFUA_5G09240), pyridoxine biosynthesis protein PyroA (AFUA_5G08090), and pyruvate carboxylase (AFUA_4G07710). BtgE is a covalently bound cell wall protein with a predicted role in degradation of glucans.

In contrast to 0 h enriched proteins, there is a much higher degree of correlation between 6 h enriched proteins and the proteins identified during early developmental stages in previous *A. fumigatus* proteomics studies (Table 2). Thus, 78 out 127 (61%) the latter were previously detected at 0 h, 4 h, 8 h and 16 h [30], including 15 and 13 proteins up-expressed at 8 h and 16 h of mycelial growth. Moreover, transcripts of 105 and 91 proteins (83% and 72%, respectively) were shown to be up-regulated at 8 h and 16 h respectively. Also, 14 and five of our germling hyphae-enriched proteins were previously identified as highly abundant in conidia and overrepresented in conidia in comparison to mycelia, respectively [6].

### Pre-septation hyphae-enriched proteins (8 h)

A total of 119 proteins were up-expressed at 8 h of fungal growth in comparison to dormant conidia (Additional file 10). Of those, 103 (87%) had an assigned GO biological function (Figure 3). At least, 26 proteins were involved in translation either as ribosomal subunits or as components of a translation elongation factor. Similar to 6 h enriched proteins, most 8 h enriched proteins were evolutionary conserved, and all but four were encoded by central chromosomal regions. All but two of the 119 proteins had orthologs in other aspergilli [3].

The list of the most abundant 8 h enriched proteins included allergen Asp F8/60 S acidic ribosomal protein P2 (AFUA_2G10100), a protein of unknown function (AFUA_1G06580), and a mitochondrial cytochrome c subunit (AFUA_2G03010). More than half of the enriched proteins were also overexpressed at 6 h and six proteins showed a pattern of exponential increase from 0 h through 8 h of fungal growth. The latter included allergen Asp F8/(AFUA_2G10100), two cell wall proteins (AFUA_4G08960 and AFUA_8G05610), nucleolar pre-rRNA processing protein Nop58 (AFUA_3G13400) and a subunit of a eukaryotic translation initiation factor (AFUA_4G03860). One GPI-anchored protein (AFUA_8G05610) is a putative adhesin, while the other (AFUA_3G00270) is cell wall glucanase BtgE. Notably, BtgE transcripts have been shown to be up-regulated during initiation of murine infection by *A. fumigatus*[15]. Additionally, six pre-septation hyphae-enriched proteins were detected previously in the secreted *A. fumigatus* proteome including Cu,Zn superoxide dismutase SodA (AFUA_5G09240) and extracellular cell wall glucanase Crf1/allergen Asp F9 (AFUA_1G16190). Similar to BtgE, SodA was previously detected in conidia and its transcripts were up-expressed in germlings during initiation of murine infection in *A. fumigatus*[15].

## Conclusions

The observed temporal expression patterns suggest that germination of *A. fumigatus* conidia involves dramatic changes in protein abundance levels. Some of the 375 identified proteins may represent novel antigens and stage-specific biomarkers of colonization, infection or treatment efficacy. Developmental stage candidate biomarkers include the following proteins: (0 h) Grg1, AFUA_6G12000; (4 h) Hsp90 binding co-chaperone Wos2 and a CipC family protein; (6 h) 40 S ribosomal protein S19 and the conserved protein AFUA_2G10580; and (8 h) telomere and ribosome associated protein Stm1 and glycine-rich RNA-binding protein. Additionally, we found that the *A. fumigatus* conidial proteome is dominated by small, lineage-specific proteins that may play key roles in host-pathogen interactions and in transmitting environmental signals that control conidial germination. Small proteins are more difficult to study than larger proteins using traditional biochemical and molecular methods. Our results show that shotgun proteomics can facilitate functional characterization of these interesting targets, which can be exploited to make the fungus more vulnerable to the host immune system.

## Methods

### *A. Fumigatus* growth and harvest

3 x 10^8^ per 100 ml of *A. fumigatus* CEA10 conidia were washed with H_2_O and inoculated into Glucose Minimal Media and incubated at 37°C at 200 rpm for 4, 6 and 8 h. For the 0 h time point, freshly harvested conidia were used. Cell wall protein extraction was conducted using a modified version of a previously described protocol [20,69]. The cells were harvested using Corning 500 ml bottle top filter and rinsed with cold sterile water and then with 10 mM Tris–HCl, pH7.5.

### Protein digestion

The frozen conidia pellet was ground to a fine powder using a mortar and pestle. Cells were re-suspended in 10 mM Tris–HCl, pH 7.5 (25 ul/mg) in the presence of a protease inhibitor cocktail (Roche, complete Mini EDTA-free Protease inhibitor cocktail). Soluble proteins, likely to be primarily of intracellular origin, were removed by washing the insoluble fraction three times with 1 M NaCl, centrifuging at 300 rpm for 10 min at 4°C between each wash. The insoluble fraction was then twice extracted for 5 min at 100°C with SDS extraction buffer (50 mM Tris–HCl, pH 7.8, 2%SDS, 100 mM NaEDTA, and 40 mM β-mercaptoethanol). The SDS treated insoluble fraction was washed three times with water and spun at 300 rpm for 5 min between each wash, followed by incubation with 30 mM NaOH at 4°C for 17 h with gentle shaking. The reaction was stopped by addition of neutralizing amounts of acetic acid. Overnight dialysis of the released proteins at 4°C was carried out. The proteins were precipitated by adding 9 volumes of 100% methanol buffer (100% methanol, 50 mM Tris HCl, pH 7.8), incubating at 0°C for 2 h, and centrifugation at 13,000 rpm for 10 min at 4°C. The pellet was washed twice with 90% methanol buffer (90% methanol, 50 mM Tris HCl pH 7.8) and air dried. The pellet was dissolved in 10 mM Tris HCl pH 7.5. The protein concentration was determined according to the method of Bradford using BIO-RAD protein assay (BIO-RAD Lab., U.K.) [70]. The ten analyzed samples contained between 35 and 70 μg protein, suggesting that this extraction procedure did not result in retention of large amounts of intracellular protein. They were processed using filter-aided sample preparation (FASP) and suitable for downstream mass spectrometric analysis [71]. In this way, in-solution digestion was carried out in the filter device, where denatured proteins were digested under the condition of maintaining the activity of the trypsin without a carboxyamidomethylation step to modify cysteine residues. The entire protein digests checked in SDS-PAGE gels for completion of digestion were analyzed by LC-MS/MS to identify *A. fumigatus* proteins.

### LC-MS/MS

LC-MS/MS analysis was performed with a LTQ ion trap mass spectrometer (Thermo-Finnigan, San Jose, CA) equipped with a Finnigan nESI source. An Agilent 1100 series solvent delivery system (Agilent, Palo Alto, CA) was interfaced with the LTQ instrument to deliver samples to a peptide trapping cartridge (CapTrap, Michrom BioResources, Auburn, CA), followed by a reversed-phase column. Peptides were eluted from the C_18_ cartridge and separated on the BioBasic C_18_ column (BioBasic C_18_, 75 μm × 10 cm, New Objective, Woburn, MA) for 85 min run [53 min binary gradient run from 97% solvent A (0.1% formic acid) to 80% solvent B (0.1% formic acid, 90% AcCN) at a flow rate of 350 nl/min.] Mass spectra were acquired in automated MS/MS mode, with the top five parent ions selected for fragmentation in scans of the m/z range 300–1,500 and with a dynamic exclusion setting of 90 s, deselecting repeatedly observed ions for MS/MS as previously provided [72].

### MS data analysis

MS and MS/MS sequences obtained from LC-MS/MS experiments were searched against the latest release of the NCBI *A. fumigatus* proteome (WGS AAHF01000001-AAHF01000019) using the search engine Mascot v. 2.3.2 (Matrix Science, London, UK). LTQ peak lists were created with Mascot Daemon using the data import filter lcq_dta.exe from XCaliber v.2.2 (Thermo electron), which coverts binary.raw files into peak list.dta files. The data were retrieved with search parameters set as follows: enzyme, trypsin; allowance of up to one missed cleavage peptide; MS tolerance ±1.4 Da and MS/MS tolerance ± 0.5 Da; no modification of cysteine and methionine oxidation when appropriate with auto hits allowed only significant hits to be reported. The protein identifications were accepted as significant when a Mascot protein score >75 and at least one peptide e-value <0.01 were reported. To accept a Mascot score between 40 and 75, a protein had to be identified as least two times with at least two peptide e-value <0.05 each. Using a randomized decoy database and a default significance threshold of 0.05 in Mascot, the false-positive rate for peptides identified by LC-MS/MS was 1.6%. Following file conversion into the ‘mzXML’ format, MS data were re-scored using the algorithms PeptideProphet™ and ProteinProphet™ [73]. The data is available in the PRIDE database [74] (http://www.ebi.ac.uk/pride) under accession numbers [19312–19315].

### Calculation of protein abundance estimates using the APEX method

The LC-MS/MS data from biological replicates (duplicates for 4 h and 6 h time points; triplicates for 0 h and 8 h time points) were combined to calculate absolute protein expression (APEX) values using a computationally modified spectral counting approach developed by Lu et al. [10] and converted into a software application by Braisted et al. termed the APEX quantitative proteomics tool v1.1 [16]. Briefly, the XML spectral data files were converted into Peptide/Protein Prophet probabilities, and O_i_ correction factors based on probability of peptide detection determined to adjust the protein quantities based on spectral counts. Default settings for peptide physicochemical properties were used to determine O_i_ values. A normalization factor of 2.0 × 10^6^ was used to convert the APEX scores into estimates of protein molecules per cell. The protein FDR was set at 1% to eliminate proteins identified at a confidence level lower than 99%. To apply a higher stringency level to the evaluation of differential protein abundances comparing the four time points, only proteins with the following filter criteria were included in the abundance analysis: (1) a total significant peptide count of at least 4 according to Mascot and APEX data and a significant unique peptide count in Mascot of at least 2 or (2) an APEX scores higher than 3,500. To identify differentially expressed proteins, Log2 ratios were used to measure relative changes in expression level at 4 h, 6 h and 8 h time points with respect 0 h. To add another level of quantification stringency, proteins were considered differentially expressed if their APEX expression values were above 3,500 and their corresponding log2 ratios were greater than 1.5 or less than −1.5.

### Prediction of signal peptide, subcellular localization and gene ontology terms

For the prediction of N-terminal signal peptides and transmembrane regions, acquired amino acid sequences of all proteins were searched with the algorithms SignalP and TMHMM (http://www.cbs.dtu.dk). For subcellular locations, a WoLF PSORT software (freely available at wolfpsort.org) was used to predict the subcellular localization. Gene Ontology (GO) terms were downloaded from AspGD (http://www.aspergillusgenome.org) [75]. The GO Slimmer tool (http://amigo.geneontology.org) was used to obtain higher level broader parent terms GO molecular function and cellular localization predictions also known as GO Slim terms.

## Abbreviations

IA: Invasive aspergillosis; FASP: filter aided sample preparation; 2-D PAGE: 2-Dimensional polyacrylamide gel electrophoresis; ESI: electrospray Ionization; APEX: absolute protein expression, also called label-free computationally modified spectral counting method; LC-MS/MS: liquid chromatography tandem mass spectrometry; iTRAQ: isobaric tagging for relative and absolute quantitation.

## Authors' contributions

MM and RP initiated and coordinated this study and contributed to the preparation of the manuscript. RP selected the proteomics approach, MS conducted the proteomics analysis. MS and NDF performed the analysis and interpretation of data and drafted the manuscript, and SH cultured *A. fumigatus* and prepared all protein extracts. RDF, SNP, WCN, SC and DP have been involved in revising of the manuscript and made contributions to the study conception and design. All authors have read and approved the final manuscript.

## Competing interests

The author(s) declare that they have no competing interests.

## Supplementary Material

Additional file 1 **Protein map providing M**_**r**_**and p*I*values for the*A. fumigatus*proteome.** Click here for file

Additional file 2 All proteins and peptides identified by LC-nESI-MS/MS on a Linear Ion Trap instrument. Click here for file

Additional file 3 Proteins identified with high confidence for at least one time point. Click here for file

Additional file 4 Cellular localizations of proteins enriched during early development. Click here for file

Additional file 5 Molecular functions of proteins enriched during early development. Click here for file

Additional file 6 Proteins expressed at all four stages during early development. Click here for file

Additional file 7 Proteins enriched in dormant conidia (0 h). Click here for file

Additional file 8 Proteins enriched in expanding conidia (4 h). Click here for file

Additional file 9 Proteins enriched in early germ tubes (6 h). Click here for file

Additional file 10 Proteins enriched in pre-septation hyphae (8 h). Click here for file
