# Supplementary figures and images for: Development stage-specific proteomic profiling uncovers small, lineage specific proteins most abundant in the Aspergillus Fumigatus conidial proteome
Source: Proteome Sci. 2012 Apr 30;10:30. doi: 10.1186/1477-5956-10-30 (PMC3424117; doi:10.1186/1477-5956-10-30)

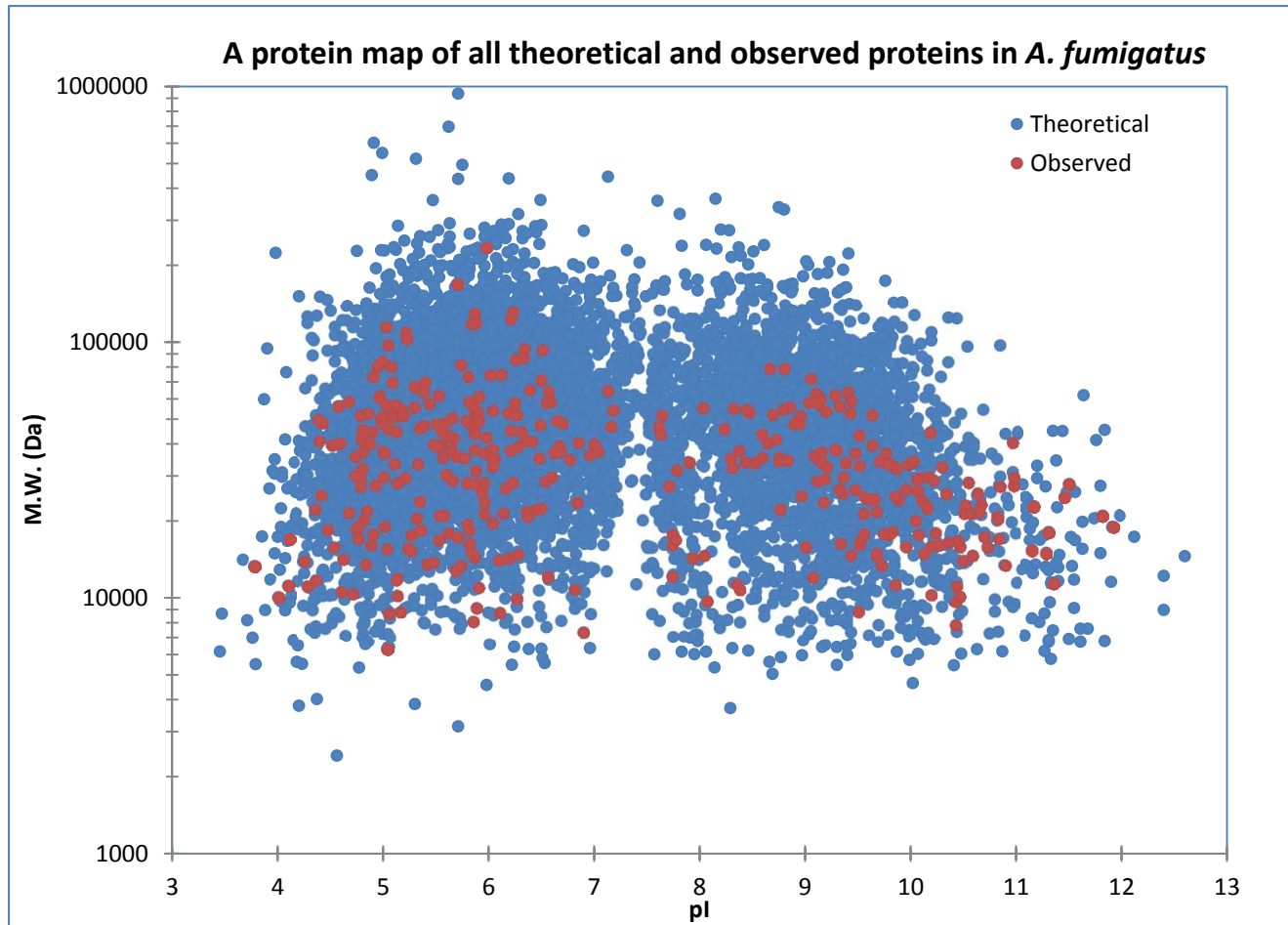

Additional Figure 1.

Supplement: Additional file 1 — Protein map providing Mrand pIvalues for theA. fumigatusproteome. [file 1477-5956-10-30-S1.pdf]
